# Chemical Composition and Functional Properties of Spray-Dried Animal Plasma and Its Contributions to Livestock and Pet Health: A Review

**DOI:** 10.3390/ani13152484

**Published:** 2023-08-01

**Authors:** Katarzyna Kazimierska, Wioletta Biel

**Affiliations:** Department of Monogastric Animal Sciences, Division of Animal Nutrition and Food, West Pomeranian University of Technology in Szczecin, 29 Klemensa Janickiego, 71270 Szczecin, Poland; katarzyna.kazimierska@zut.edu.pl

**Keywords:** amino acids, aquaculture, blood products, domestic animals, feed additives, pet food

## Abstract

**Simple Summary:**

In recent years, the livestock industry has faced challenges related to economic issues, environmental concerns, and climate change. To address these challenges, researchers are exploring new feed and feeding practices. One such approach is the utilization of spray-dried animal plasma (SDAP), which is derived from collected animal blood and processed using spray drying to preserve its functional properties. It contains essential nutrients, immune-boosting substances, and growth factors. It has been shown to improve growth, intestinal development, and health in weaning piglets, as well as enhancing growth and disease resistance in poultry. SDAP is also used in pet nutrition, contributing to improved pet food texture, digestion, and reduced fecal output. However, there are limitations, including cost and safety concerns. This review aims to provide an overview of the chemical composition of SDAP from different animal species and its role in promoting performance and health benefits for farm animals, aquaculture, and pets.

**Abstract:**

Spray-dried animal plasma (SDAP) is a functional ingredient derived from healthy animal blood, used as a nutritional additive in livestock and pet nutrition. SDAP is rich in macronutrients, micronutrients, and bioactive compounds such as immunoglobulins, albumin, growth factors, peptides, transferrin, and enzymes. This review focuses on the chemical composition of SDAP from porcine, bovine, and poultry sources, including protein quality and mineral profile. SDAP enhances performance and health in monogastric farm animals, aquaculture, and pets. It promotes growth rates and feed intake due to its high digestibility and superior amino acid profile compared to other protein sources. In pigs, SDAP’s positive effects stem from tissue-specific actions in the gastrointestinal tract, impacting digestion, immunity, and barrier function. For poultry, SDAP shows promise as a substitute for antibiotic growth promoters, particularly in chick starter diets. SDAP contains functional proteins that regulate immune response, enhance intestinal health, and aid in stress conditions. It is also used as a binder in pet food, providing high protein content and other desirable properties. SDAP meets the dietary requirements of carnivorous pets, appealing to owners seeking animal-derived protein sources. Additionally, SDAP may help prevent cognitive impairment in senior dogs and cats.

## 1. Introduction

In recent decades, notable advancements have been made in modern livestock farming, particularly in the realm of feed and feeding practices. However, the convergence of present global economic issues, the ongoing reputational crisis surrounding livestock production, the hurdles posed by climate change, and the associated emissions and resource limitations linked to this industry have engendered significant challenges [1,2]. These challenges necessitate innovative solutions in the agricultural and feed industry sectors, particularly concerning the assessment and utilization of by-products, especially liquid ones [3,4]. One of these products is spray-dried animal plasma (SDAP) derived from preserved blood through a process called spray drying and is an important nutritional additive in livestock and pet animal nutrition. SDAP is a diverse mixture of functional components, including immunoglobulins, albumin, fibrinogen, lipids, growth factors, peptides, and other factors, exhibiting biological activity [5].

Previous research has demonstrated the positive effects of SDAP inclusion in weaning piglets’ diets, leading to improved growth performance, intestinal development, overall health, and the mitigation of enteric infections [6,7,8,9,10]. SDAP, as a relatively novel biogenic, has demonstrated successful utilization in recent years for combating such infections [11]. A comprehensive meta-analysis revealed significant improvements in average daily gain (ADG) and average daily feed intake (ADFI) in piglet diets with SDAP, showing increases of +26.8% in ADG and 24.5% in ADFI during the initial two weeks following weaning [12]. At the time, there were no other feed ingredients or additives that had such large effects. Similarly, dietary inclusion of SDAP is expected to have a positive impact on growth, mineral retention, gut health, and disease resistance in poultry, especially when they are raised under unsanitary or intensive rearing conditions that expose them to various stressors [13,14]. Also, the use of SDAP in calves has been studied and shown to have positive effects on health, growth, and intake. The results of one study showed that calves fed a diet with the addition of 30 to 60 g/day of SDAP to milk replacer had fewer days with diarrhea, lower use of electrolytes, and improved body weight gain [15]. Similarly, in another study, male Holstein calves fed milk replacers containing 5% SDAP had reduced morbidity and mortality compared to those fed milk replacers without SDAP [16].

A global movement aims to promote responsible antibiotic use in livestock farming to combat antibiotic-resistant pathogens that pose a threat to human health. Swine producers are exploring alternatives to antibiotics, and experiments with pigs have investigated the positive outcomes of SDAP, both with and without growth-promoting antibiotics [17,18,19]. Similarly, in poultry nutrition, results of studies evaluating the impact of the dietary inclusion of SDAP highlight its potential as an effective and sustainable alternative to traditional antibiotic use in broiler diets [20,21]. Research on ruminants indicates that SDAP can be a viable alternative to antibiotics in calf milk replacer applications as well, especially in managing enteric challenges [15,22]. In calves infected with *Cryptosporidium parvum*, bovine serum concentrate reduced fecal losses and returned villus surface area to normal values [23]. Also, in aquaculture, SDAP additive can improve the health conditions and enhance the disease resistance of aquatic animals [24]. Most studies have not reported interactions, suggesting an additive effect of SDAP to growth-promoting antibiotics. Therefore, incorporating SDAP into animal feed can enable producers to reduce or eliminate the need for antibiotics, promoting more sustainable and responsible animal farming practices.

In companion animal nutrition, SDAP is used in wet and dry dog foods, offering technological properties such as gel strength, water retention, and fat emulsion capacities. Texturing capacity of animal plasma concerns heat-induced formation of a stable gel. The proteins lose their structure and bind both water and fat, thus being able to form a network with pieces of foodstuffs. The inclusion of SDAP increased the texture and reduced water loss from chunk and loaf pet foods, compared with the other binders tested [25]. Studies have indicated that SDAP supplementation improves digestion and reduces fecal output in dogs [26].

Despite these benefits, there are limitations to the widespread use of SDAP in animal diets. Cost considerations may lead feed producers and breeders to opt for cheaper alternatives with similar properties, such as fish meal or whey powder. However, recent research has highlighted the economic efficiency of incorporating spray-dried bovine hemoglobin powder in fish diets [27]. Secondly, although the significant advantages of SDAP on animal health and performance have been extensively documented, concerns may arise regarding its safety, considering its origin from abattoir-collected animal blood, especially in the context of emerging or re-emerging diseases within animal populations [28].

The objective of this review is to discuss the results of available studies on the nutritive value of spray-dried animal plasma obtained from different animal species (porcine, bovine, and poultry) including protein quality and mineral profile, the use of this ingredient in promoting overall performance, and health benefits for livestock animals, aquaculture, as well as pets.

## 2. Production of Spray-Dried Plasma

**Figure 1:**
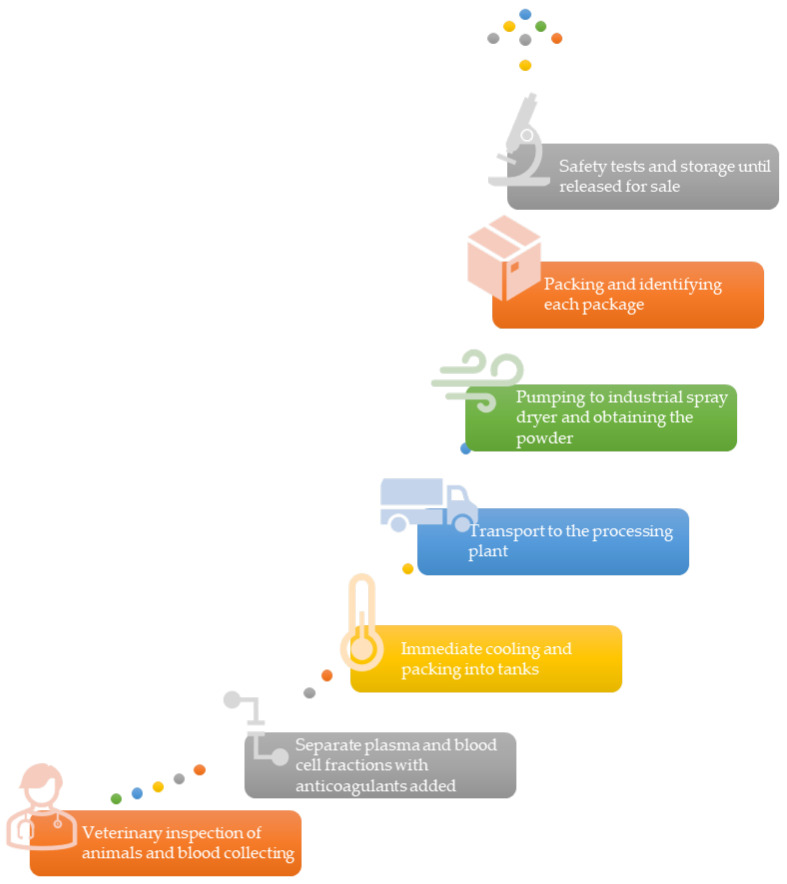
Diagram of the most important steps of spray-dried plasma production (adapted from [11,17]).

Spray-dried plasma is produced from porcine (SDPP), bovine (SDBP), or poultry (mainly chicken, SDCP) blood and is commonly used in human food and animal feed. The commercial production of SDAP adheres to strict good manufacturing practices [29] to ensure the production of a safe and high-quality product. SDAP is produced from fresh animal blood from veterinary-inspected animals (Figure 1) and determined fit for slaughter for human consumption [30,31]. Blood is only drawn when the carcass is intact, thus minimizing contact with other tissues. Furthermore, the blood collection system is separate from the rest of the carcass processing chain. The raw material, which meets bacteriological requirements, is treated with an adequate quantity of anticoagulant. Subsequently, it is transported in a refrigerated truck, maintaining a temperature range of 3–5 °C. The blood is then drawn from the container using a membrane pump and directed towards a disk-type centrifuge. Centrifugation, a widely utilized method, is employed to separate the blood into two components: plasma (displaying an amber color) and red hemoglobin (forming an erythrocyte slurry). By rotating at 700 rpm, the centrifuge effectively segregates the blood into plasma and hemoglobin, which are subsequently transported to buffering containers. After separation, the plasma is concentrated or not concentrated and refrigerated at 4 °C. Then the plasma is transported to the processing plant in cleaned, sealed dedicated tankers. Storage tanks at abattoirs or spray-drying plants are cleaned and sanitized after emptying [32].

At the processing plant, the plasma is pumped into an industrial spray dryer, which rapidly dries the concentrated liquid plasma into powder (Figure 1). In most cases, prior to drying, plasma is concentrated by evaporation or membrane filtration [33]. Spray drying consists of atomization of a liquid feed material into a stream of heated air resulting in rapid desiccation. Plasma is spray dried under high pressure at high temperatures (80 °C throughout its substance) to convert it to powder. This is designed to maintain the biological activity of the proteins, primarily albumin and globulins. Notably, immunoglobulin G (IgG) is the prevailing antibody type, and its biological activity is effectively preserved throughout this process. It is expected that immunoglobulins in SDAP can optimize performance in fed animals through passive immunity [17,34].

To avoid any changes in plasma color, it is necessary to apply separate spray dryers for erythrocytes and plasma. The spray-drying process is closely monitored and controlled using standard operating procedures, to ensure quality and safety. A computer-controlled system is employed to oversee the process, and continuous monitoring and recording are carried out. After spray-drying, each batch of the product is meticulously identified on the packaging. Prior to being released for sale, rigorous quality control and safety tests are conducted to guarantee that the product meets the required standards. The product batch number allows traceability of distribution to customers. The detailed SDAP obtaining process and a comprehensive specification of each of the key safety steps in production are widely described by Blázquez et al. [28] with special focus on a new redundant pathogen inactivation step, UV-C irradiation. The application of non-thermal ultraviolet radiation at 254 nm wavelength (UV-C) results in the disruption of cellular transcription and replication in bacteria, viruses, and molds, leading to their death [35,36]. This technique is commonly employed for industrial disinfection of water, milk, and fruit juice. Importantly, the use of UV-C for disinfection has shown limited adverse effects on the nutritional quality of the treated liquids [37,38,39].

To enhance storage and transportation efficiency, the liquid plasma is transformed into a powder form through the process of spray drying. Spray drying offers several advantages, including straightforward operation, rapid drying time, cost-effectiveness, and suitability for large-scale continuous production. The conversion of the protein into powder eliminates various drawbacks associated with its liquid form, such as perishability and challenges in storage and transportation [40].

### Safety

As SDAP is a blood-derived feed ingredient, its safety is frequently tested, especially during periods of emergence or re-emergence of animal diseases in different regions of the world. The spray-drying process used to manufacture SDAP involves sudden changes in temperature and pressure, causing rapid evaporation of water, which is an effective technology for inactivating important pathogens in the swine and bovine industries [41]. The low moisture (<9%) and very low water activity of SDAP significantly reduce pathogen survival, especially for bacteria and enveloped viruses during longer storage periods [42]. Hurdles in SDAP production in terms of inactivating potential viral contamination include, in addition to spray drying (80 °C throughout the substance), treatment with ultraviolet (UV) light (3000 J/L) [28]. According to Sampedro et al. [42], as an added safety measure, many manufacturers adopt a practice of packaging and storing SDAP at room temperature (above 20 °C) for a minimum of 14 days before its release for sale. This storage approach has been demonstrated to effectively deactivate specific pathogens that are susceptible to dry environments and mild temperatures, such as the porcine reproductive and respiratory syndrome virus (PRRSV), porcine epidemic diarrhea virus (PEDV), and coronaviruses in general [42,43]. Furthermore, several studies have indicated that commercially available SDAP, when orally consumed, does not transmit either porcine parvovirus (PPV) or porcine circovirus type 2 (PCV2) [41,44,45,46], which are the two most heat- and chemical-resistant swine viruses. Also, RNA of PEDV present in commercial SDAP was found to be non-infectious for naïve pigs [47]. Experimental and epidemiological evidence usually demonstrates that SDAP does not spread diseases [41,42,43,44,45,46,47]. The spray-drying process has been shown to be effective in inactivating infectious bovine leukemia virus (BLV) in colostrum [48]. Also, in humans, studies assessing the acceptability and safety of SDAP supplementation show that it may increase the fractional absorption of dietary lipid and of total energy in malnourished children, and there was no evidence of any adverse effects of bovine serum concentrate [49].

Spray-dried plasma proteins have also been demonstrated to be a safe and high-quality feed ingredient in ruminant studies. Studies investigating the effects of SDAP supplementation on dairy cows [50,51] demonstrated a positive influence on milk production and composition without negatively affecting reproductive performance (e.g., pregnancy rate and conception rate). Notably, SDAP-fed cows exhibited slightly higher milk yield and milk fat content, with higher SDAP levels (400 g/day vs. 100 g/day) leading to greater milk yields [50]. However, it is essential to consider that these studies were conducted in the United States of America. In the European Union, the utilization of blood products derived from both ruminant and non-ruminant animals is prohibited in ruminant nutrition [52].

A recent study conducted by Blázquez et al. [53] aimed to assess the prevalence and magnitude of potential viral contamination in commercially harvested porcine plasma. The study spanned a period of 12 months and involved eight spray dryers located worldwide. The findings revealed that the virus levels detected in samples containing viral DNA/RNA were relatively low compared to the levels observed during the peak viremia of an infection for all viruses. Additionally, the levels were also below the minimal infectious dose for each respective virus, as reported in other studies [54,55,56,57]. The estimated level of viral contamination in commercially collected porcine plasma varied, with most viruses showing levels below 2.0 log_10_ TCID_50_. However, sporadic instances of swine influenza virus (SIV) contamination were observed, reaching levels as high as 4.5 log_10_ TCID_50_/g of liquid plasma.

## 3. Spray-Dried Animal Plasma Chemical Composition

Soluble concentrated protein is a raw material that has long been used for farm animals as a high-protein feed material, and recently, it is also an ingredient used in pet foods. Blood plasma, as a functional protein source, contains more than 697 proteins in its composition [58]. This complex fluid encompasses a diverse range of components, including proteins, cytokines, growth factors, hormones, bioactive peptides, and amino acids. The inclusion of SDAP in animal diets has been associated with numerous health benefits at both systemic and various mucosal levels, attributed to these constituents [11]. Spray-dried animal plasma, an abattoir by-product utilized in animal nutrition, is favored for its exceptional amino acid profile and an impressive digestibility rate of approximately 99%. SDAP contains a water content lower than 10 g/100 g, 7–11 g/100 g of ash, and 70–80 g/100 g of protein (Table 1 and Table 2). SDPP had the highest crude protein (CP) concentration (75.1%) among the five feedstuffs tested by Zhang et al. [34] (albumen powder, 73.2%; fish meal, 67.2%; dried porcine solubles, 51.7%; and spray-dried egg, 42.8%).

The protein composition of SDAP is 50–60% albumin, 40–50% globulins, and 1–3% fibrinogen [59]. Although the protein content of SDAP is lower than that of casein (96 g/100 g) [60], the quality of this protein (based on amino acid composition) is relatively high. Regarding the contents of essential amino acids (EAAs), SDAP is superior to soybean protein [60]. SDAP contains a relatively high concentration of Lys, Trp, and Thr when compared to isolated soy protein, maize gluten meal, and soybean meal [60], but at the same time, it has relatively low concentration of Met. The amino acids limiting the quality of the protein are mainly Ile and Met + Cys. As shown in the presented work, based on literature data for amino acids with reference to the whole egg protein (WEP) standards, the first limiting amino acid in SDPP and SDCP is Ile (Table 1) and in SDBP they are sulfur amino acids (Met + Cys). In the case of SDPP and SDCP, the second limiting amino acid was found to be sulfur amino acids, and in SDBP, respectively, the opposite, i.e., Ile (Table 1). Our own calculations confirmed the literature data, in which Ile is usually the limiting amino acid in blood proteins. Jamroz et al. [61], evaluating the chemical composition of spray-dried porcine blood by-products and bone protein hydrolysate, stated that the best composition, considered from the point of view of limited amino acids in the diets of monogastric animals (such as Met + Cys, Lys, Trp, and Thr), was found in dried plasma rather than blood cell or bone protein hydrolysate. Zhang et al. [34] proved that concentrations of the essential amino acids (EAAs) were greater in SDPP than the other protein sources (albumen powder, fish meal, dried porcine solubles, and spray-dried egg) except for Arg and Ile. SDPP was the highest in both Thr (4.1%) and Trp (1.5%). In turn, compared to *Arthrospira platensis*, the most cultured microalga worldwide [62] used as a supplement in animal diets, SDAP has a lower EAA content. However, SDAP contains more His, Lys, and Trp than spirulina [63,64]. Whereas in comparison to whey protein concentrate, which had relatively high Lys, Ile, Leu, Thr, and Trp content [65,66], SDAP was richer in Arg, His, Phe, and Val.

In a study evaluating the chemical and biological characteristics of spray-dried plasma protein collected from various locations around the world, no differences were found in SDAP protein content when plants manufacturing similar products were compared [67].

Overall, blood products are a good source of nutrients, especially due to the high content of essential amino acids, but also due to the high content of minerals, especially iron. SDAP contains a significant amount of minerals expressed in the form of crude ash. The literature values for SDAP ash content range from 7.26% in spray-dried bovine plasma [68] to an average of 11.09% in chicken plasma [8,69,70,71] (Table 2). Spray-dried porcine plasma contains about 10 mg of Fe [61] (Table 2), which is similar to the Fe content in spray-dried egg [72,73]. However, SDAP is a dietary ingredient richer in iron than fish meal (<1 g/100 g of Fe) [74,75] and whey powder (1–2 mg/100 g of Fe) [76,77]. SDAP also contains more Zn than whey protein concentrate powder, 5 mg/100 g (on average; Table 2) and 0.33 mg/100 g, respectively [78]. However, compared to the increasingly popular feed ingredient *Arthrospira platensis*, SDAP has less Ca, P, K, Mg, and also Fe, but more Na and Cu [79,80,81]. Minerals present in SDAP are very digestible and soluble and, consequently, highly available for animals. The addition of SDAP to a recipe of wet cat food led to enhancements in the apparent digestibility of crude ash, calcium, and phosphorus [82]. This increase in phosphorus digestibility aligns with findings from studies conducted on pigs, where diets containing SDAP also exhibited high phosphorus digestibility [83].

**Table 1:** Crude protein, amino acids, and nutritive value of protein of spray-dried plasma with the division into species of animals from which the plasma was obtained.

Item	Spray-Dried Porcine Plasma	Spray-Dried Bovine Plasma	Spray-Dried Chicken Plasma
Crude protein (g/100 g)	78.74	75.21	76.74
Essential amino acids (g/100 g CP)			
Arginine, Arg	3.77	4.15	4.48
Histidine, His	2.20	3.84	2.77
Isoleucine, Ile	2.14 ^5^	3.13 ^6^	2.68 ^5^
Leucine, Leu	6.29	9.72	7.51
Lysine, Lys	5.84	8.34	5.98
Methionine, Met	0.69	0.93	1.38
Methionine + Cystine, Met + Cys	2.37 ^6^	2.37 ^5^	3.75 ^6^
Phenyloalanine, Phe	3.42	5.38	4.16
Phenyloalanine + Tyrosine, Phe + Tyr	6.45	9.57	7.32
Threonine, Thr	4.49	6.45	4.50
Tryptophan, Trp	1.03	1.54	1.27
Valine, Val	4.01	6.87	4.81
Non-essential amino acids (g/100 g CP)			
Alanine, Ala	3.41	5.15	4.86
Aspartic acid, Asp	6.50	10.25	7.70
Glutamic acid, Glu	9.08	13.89	11.76
Glycine, Gly	2.33	3.50	3.20
Proline, Pro	3.81	4.27	3.84
Serine, Ser	4.00	6.09	5.08
Nutritional values			
Ʃ AA ^1^	67.72	99.13	81.51
Ʃ EAA ^2^	38.57	55.98	45.07
CS ^3^	39.63	41.58	49.63
EAAI ^4^	65.36	82.12	76.53
References	[8,25,34,61,84]	[68,85]	[8,69,70,71]

^1^ AA—amino acid; ^2^ EAA—essential amino acid; ^3^ CS—chemical score calculated on the basis of whole egg protein (WEP) standards; ^4^ EAAI, essential amino acid index; ^5^ the first limiting amino acid; ^6^ the second limiting amino acid.

**Table 2:** Crude ash and mineral composition of spray-dried plasma with the division into species of animals from which the plasma was obtained.

Item	Spray-Dried Porcine Plasma	Spray-Dried Bovine Plasma	Spray-Dried Chicken Plasma
Crude ash (g/100 g)	9.10	7.26	11.01
Macroelements (g/100 g)			
Ca	0.18	0.08	0.22
P	0.43	0.10	0.62
K	0.13	0.34	nd ^1^
Na	5.23	5.50	nd
Cl	2.19	6.80	nd
Mg	0.15	0.02	0.04
Microelements (mg/100 g)			
Cu	5.00	1.5	nd
Fe	9.98	nd	nd
Mn	1.10	nd	nd
Zn	8.00	1.5	nd
References	[8,25,34,61,84]	[68,85]	[8,69,70,71]

^1^ nd—no data.

## 4. The Use of SDAP in Swine and Piglet Nutrition

SDAP was first proposed as a protein source for use in piglet feed in the late 1980s [86,87,88]. Since then, many studies have demonstrated an improvement in piglet performance with its use.

SDAP was shown to produce a positive response in growth rates and feed intakes due to its high digestibility compared with diets containing other protein sources. Zhang et al. [34] compared various protein sources, including spray-dried egg (SPE) and albumen powder (AP), with SDPP, dried porcine solubles (DPSs), and fish meal (FM). Among these sources, SDPP, with 20% additive, demonstrated the highest competitiveness as a protein component, offering digestible nutrients, improved amino acid utilization, and abundant bioactive substances like immunoglobulins and lysozyme. These substances supported immune regulation and promoted a healthy intestinal microflora. SDPP showed apparent total tract digestibility (ATTD) of 84.05% for gross energy, 85.09% for dry matter (DM), and 76.85% for crude protein (CP).

Weaning stress and milk withdrawal are critical stages in pig production that can result in various internal disorders, including intestinal disorders, growth checks, and poor immunocompetence. High-quality protein diets, like those with SDAP added, are required to counteract the harmful effects of weaning stress. Edwards et al. [6] reported that a 5% inclusion of SDPP in piglets’ diet enhanced their growth performance, with better weight gain, feed disappearance, and feed conversion efficiency compared to 3.5% yeast protein meal. Torrallardona [89] conducted a comprehensive study reviewing data from 75 trials across 43 publications involving over 12,000 piglets. The study suggested that an optimal 4% to 8% inclusion of SDPP in piglets’ diet during the two-week pre-starter period maximized productivity and improved immunity. More specifically, the study observed that an inclusion level of 6% of SDPP resulted in better performance compared to lower inclusion levels such as 2% and 4% [90], or compared to inclusion levels of 2%, 4%, and 8% [91]. Similarly, higher performance was observed with an inclusion level of 6% compared to 3% SDPP [92,93], 5% compared to 2.5% SDAP [94], and 7% compared to 3.5% SDAP [95]. However, lower dosage response studies demonstrated that satisfactory performance could still be achieved at inclusion levels of around 3% [17,94]. It is important to note that higher doses of SDAP can result in nutrient imbalances, such as methionine, isoleucine, or salt, which can reduce productive responses.

In a study conducted to investigate the effects of SDPP or SDCP supplementation in diets without the inclusion of antibiotics and zinc oxide (ZnO) on growth performance in early weaned piglets [8], results showed that piglets fed a 5% SDPP diet had higher average daily feed intake (ADFI) than other groups. As a result, ADG and body weight (BW) at day 14 were significantly higher in the SDPP group than in the control and SDCP groups. A meta-analysis of 143 experiments demonstrated that weaned pigs fed diets with SDAP had better ADG, ADFI, and feed-to-gain ratio (FG) regardless of the presence or absence of antibiotics in the diet [89].

SDAP, containing beneficial fractions like fibrinogen, albumin, globulin, and immunoglobulins that help improve the growth efficacy of piglets [7], is a relatively expensive feed ingredient, leading to a search for alternatives. SDPP-fed pigs (4% and 6% inclusion of SDPP) grew faster than pigs fed soy protein concentrate during the initial 10 days after weaning, and accordingly, feed efficiency was higher in SDPP-fed pigs [96,97]. Additionally, SDPP (4.5% inclusion) promoted higher weight gain compared to fish meal [9]. In a recent study conducted by Cho et al. [98], the effect of including full-fatted mealworm larvae (FFML) or hydrolysate mealworm larvae (HML) from *Tenebrio molitor* as a protein source in the diet of nursery pigs was examined as a potential substitute for SDPP. The findings indicated that the inclusion of FFML or HML in the diet did not result in improved performance compared to a standard control diet containing SDPP. Throughout the trial, the growth performance, nutrient digestibility, and fecal score of the animals were comparable between the FFML or HML diet and the control diet containing 3% or 6% SDPP. Among various insect species [99,100], *T. molitor* has been suggested as an acceptable alternative to SDPP.

However, beneficial effects of SDAP, especially on ADG and ADFI, were observed primarily during the first week after weaning [6,101]. The beneficial effects of SDAP decrease with an increase in weaning age. In a study conducted by Castelo et al. [102], the researchers aimed to investigate the effects of a prolonged feeding program with high inclusions of SDAP (0.0%, 3.0%, 6.0%, or 9.0%) after weaning on the growth performance and disease resistance of pigs. The hypothesis was that feeding pigs with increased levels of SDAP for 28 days after weaning (21 days) would result in improved growth performance and enhanced resistance to diseases. The results of the study demonstrated that the prolonged use of SDAP in the post-weaning diet had a positive impact on the growth performance of pigs. Additionally, when the pigs were challenged with *Escherichia coli*, bacterial shedding was reduced in pigs fed with increased levels of SDAP. Notably, positive growth outcomes observed in the pigs fed with the higher levels of SDAP persisted even after the withdrawal of SDAP from their diet.

Increased growth performance and food intake in pigs fed with SDAP can be explained by tissue-specific actions, especially within the gastrointestinal tract, which is responsible for a multitude of digestive and immune functions that depend upon the balanced interaction of the intestinal microbiota, diet, gut barrier function, and mucosal immune response. Generally, intestinal morphology is an important factor reflecting intestinal development. The increased villus height affected the nutrient absorption capability in the intestine as it increased the absorptive and surface area [103].

In a comprehensive review conducted by Petschow et al. [104], beneficial effects of animal protein isolates on intestinal integrity and reduced permeability to pathogens were demonstrated. Early weaning often leads to villus atrophy and crypt hyperplasia [105,106]. However, research conducted by Owusu-Asiedu et al. [107] revealed that pigs fed diets supplemented with SDAP exhibited increased villus height. This finding suggests that the inclusion of SDAP in the diet can help mitigate the negative effects of early weaning by promoting the maintenance of proper villus structure in the intestine. Similarly, the addition of SDCP in pig diets had comparable effects and promoted small intestine morphology [108]. In neonates with intrauterine growth retardation (IUGR), 5% SDPP in the diet increased ileal villus height and absorptive capacity, which was further supported by higher plasma concentrations of D-xylose, suggesting improved absorptive function in weanling pigs as a result of SDPP supplementation. On the other hand, in a study by Castelo et al. [102], no significant effects of SDAP feeding programs on villus height, crypt depth, or their ratio in jejunal sections of pigs were observed at 49 days of age. However, the authors concluded that this lack of effect could be attributed to the fact that the pigs had already recovered from gut disturbances approximately three weeks after the *Escherichia coli* challenge. It should be noted that the impact of SDAP on gut health was evident when the measurements were taken at an earlier stage, specifically between 28 and 35 days of age.

Intestinal crypts are invaginations of the epithelium around the villus and are lined by epithelial cells that secrete enzymes. The bases of the crypts are constantly dividing to maintain the structure of the villus. Boyer et al. [109] conducted a study to investigate the effects of different levels of SDAP inclusion (2.5% or 5%) in the diet of pigs challenged with *Salmonella typhimurium*. The researchers found that the inclusion of 5% SDAP in the nursery diet for 14 days resulted in increased crypt depth in the intestine. Increased crypt depth is commonly associated with intestinal injury, but it can also be seen as an indicator of epithelial repair processes. Therefore, the observed increase in crypt depth in pigs fed the 5% SDAP nursery diet may suggest enhanced epithelial renewal, which could potentially be beneficial during the later stages of recovery from *S. typhimurium* infection. These findings suggest that early dietary supplementation with SDAP can influence the intestinal immunological and epithelial responses to an *S. typhimurium* challenge, even after SDAP has been removed from the diet.

Spray-dried plasma has been suggested to offer beneficial effects due to its content of immunoglobulins [87], and for this reason, some SDAP sources have a standardized IgG content. Additionally, plasma sources enriched with specific immunoglobulins have been obtained from pigs vaccinated against specific pathogens, known as spray-dried immune porcine plasma (SDIPP).

Piglets, particularly after weaning at around 21 days of age, have limited immune competence due to weaning stress and the sudden cessation of maternal milk intake [97]. Additionally, during the first week after weaning, piglets exhibit low feed intake [110]. To address these challenges, diets rich in highly palatable and easily digestible animal proteins are being developed to meet the nutritional requirements and support the development of the digestive and immune systems in newly weaned piglets. One such protein source is SDAP, which can provide an adequate intake of IgG, a key component of plasma. IgGs play a crucial role in growth performance, while immunoglobulin A (IgA) is important for mucosal surfaces [7,111]. Diets containing SDAP have been shown to increase serum levels of IgA and IgG in pigs [97]. Edwards et al. [6] found increased serum IgG concentration (5.08 mg/mL) at 28 days to (9.24 mg/mL) 68 days of age in piglets fed 5% SDPP.

The exact mechanisms by which SDAP affects serum IgA and IgG concentrations in piglets around 3–4 weeks of age are not fully understood, as it is unlikely that the immunoglobulins present in SDAP are absorbed through the intestinal wall at this stage [112]. However, the IgGs present in SDAP can interact with the intestinal microbiota and modulate the host immune response [111], leading to the continuous production of IgA and IgG, even after the absorption of these immunoglobulins into the bloodstream is hindered by incomplete gut closure during weaning.

One of the most recent studies [113] investigated the benefits of feeding SDPP to pigs infected with African swine fever virus (ASFV). Pigs fed an 8% SDPP-enriched diet showed delayed transmission and reduced virus load compared to conventionally fed pigs. Enhanced T-cell priming after ASFV exposure was considered a possible mechanism for these effects. In another part of the study [114], pigs inoculated with the BA71∆CD2 ASFV vaccine prototype were fed either a conventional diet or a diet with 8% SDPP. The SDPP-fed group exhibited better protection against ASFV, showing no fever or detectable virus during the post-exposure period. The study suggests that SDPP can be used as a health management tool to enhance vaccine efficiency.

SDAP can enhance intestinal health through various mechanisms, including direct influence on the immune inflammatory response and immunomodulation at local or systemic levels, as well as indirect modification of microbial populations. Several review papers have summarized the modes of action of SDAP in more detail [5,11,115]. Regardless of the specific mechanism, SDAP was found to exert anti-inflammatory effects, resulting in improved intestinal health characterized by reduced permeability, increased nutrient transport, and systemic immunomodulation, ultimately enhancing animal performance.

## 5. Spray-Dried Animal Plasma in Poultry Nutrition

The presence of antibiotic-resistant bacteria in the gastrointestinal tracts of poultry and livestock has led to increased governmental and consumer pressure on the food animal industry to stop using antimicrobials in animal feed. As a result, research efforts have focused on identifying alternative nutritional supplements that can replace antibiotics while maintaining optimal production rates. One such ingredient reported in the literature is SDAP, which is considered a potential alternative to antibiotic growth promoters in poultry feed.

SDAP is used as a highly digestible protein source in poultry diets. Its high apparent metabolizable energy and standardized ileal amino acid (AA) digestibility, even in young chicks (7–10 days old), make it a particularly beneficial ingredient for inclusion in chick starter diets [116].

The use of SDAP in poultry diets has been limited due to cost considerations. However, the reported benefits of incorporating plasma, in terms of enhanced growth and feed conversion, suggest that it could be a valuable component in poultry farming. Research by Henn et al. [117] demonstrated that SDAP supplementation at a level of 1.5–3% improved the performance of broilers, particularly during the starter phase when birds were exposed to challenging conditions, such as the reuse of litter from a previous flock affected by coccidiosis. Similarly, Beski et al. [118] fed broilers with SDAP at levels up to 2% during the starter phase and observed improved feed efficiency that persisted throughout the grower and finisher phases, even after SDAP was no longer included in their diet. Furthermore, chicks that were offered SDAP-containing starter diets had longer villi, deeper crypts, and a lower ratio of villus height to crypt depth than the control group at 24 days of age. Beski et al. [119] found beneficial effects, including improved BW and feed conversion ratio, associated with feeding dietary inclusion levels of 1 or 2% during the first 10 days after hatching. The authors concluded that it would be more economical to use a low inclusion level of SDAP over a longer period of time to achieve the same benefits.

In broiler trials conducted under challenging conditions, SDAP addition showed benefits. In one study with two experiments, broilers raised on soiled litter exhibited improved growth rate, feed conversion, breast-meat yield, and flock uniformity when fed with 2% SDAP from 1 to 42 days of age [120]. Another study focused on broilers with necrotic enteritis, where SDAP was incorporated into the diet at levels of 1% during the starter phase, 0.5% during the grower phase, and 0.25% during the finisher phase. The findings revealed improvements in growth rate, feed intake, feed efficiency, and livability compared to the control group [121]. When broiler chickens were challenged with *Salmonella sofia* [122], birds fed diets containing SDAP had significantly higher BW, but this was only significant in the starter and grower phases. In a study in which broiler chicks were given bacitracin methylene disalicylate (BMD) antibiotic- or SDAP-supplemented (3%) diets and orally challenged with *Salmonella Enteritidis* (SE), it was reported that the SDAP diet mitigated the adverse effects of the SE challenge on broiler growth performance up to 2 weeks of age [123]. Furthermore, in high-temperature conditions (heat stress), partially feeding with SDAP helped mitigate adverse effects such as reduced body weight, feed intake, bone strength, increased feed conversion, and gut permeability [124].

The poultry industry recognizes the importance of implementing nutritional strategies that enhance the immune system, improve intestinal integrity and functionality, and enhance tolerance to stress and disease challenges. SDAP contains functional proteins such as immunoglobulins, albumin, growth factors, and biologically active peptides that can modulate the immune response and improve intestinal health. Incorporating a functional ingredient like SDAP can help maintain intestinal barrier integrity and improve immunological performance under varied stress conditions. When the immune system is activated in response to stress, pro-inflammatory cytokines are released, leading to an inflammatory response. This immune-mediated inflammation can disrupt normal growth and metabolic processes [125]. The activation of the immune system increases the demand for nutrients to support immune responses [126], potentially limiting the availability of nutrients for productive functions such as growth and performance. Therefore, enhancing the effectiveness of the immune response by promoting the restoration of immune system homeostasis and preserving the structural integrity of the intestinal barrier is crucial. By facilitating faster stabilization of the immune system activation and redirecting nutrient utilization back to productive functions, poultry can recover more quickly from stress-induced immune activation and minimize negative impacts on growth and performance.

The gastrointestinal tract is particularly vulnerable to heat stress, as it can disrupt the expression of tight junction protein genes, leading to increased permeability and chronic systemic inflammation [127,128]. The measurement of fluorescein isothiocyanate dextran (FITC-d), a small molecule with a molecular size of 3–5 kDa that is normally not absorbed by the gastrointestinal tract, had been identified as a reliable biomarker for assessing gut permeability in poultry [129]. Studies have shown that the addition of SDAP to the diet of heat-stressed chickens reduced the serum concentrations of FITC-d, indicating an improvement in intestinal barrier function and gut integrity [124]. This reduction in FITC-d levels suggests a decrease in chronic systemic inflammation associated with increased gut permeability. Early consumption of 1% SDAP in broilers also improved intestinal health and feed intake, with lasting positive effects [121]. Feeding SDAP to broilers resulted in better overall health with reduced coccidia lesions and other pathologic alterations in the small intestine and cecum as observed in a study conducted by Belote et al. [130]. Additionally, SDAP-fed birds, regardless of good or bad management practices, had lower histopathologic alterations in the ileum. Another study [131] reported the efficacy of dietary SDAP at 30 g/kg in a broiler chick diet or BMD antibiotic (at 0.055 g/kg) in improving feed conversion ratio, maintaining intestinal villi renewal, and increasing jejunal goblet cell density. However, a separate study [132] evaluating the effects of dietary inclusion of SDPP on broilers found improved growth performance, apparent total tract digestibility (ATTD) of protein, nitrogen retention, and energy retention efficiency during the starter period. Nonetheless, these improvements could not be attributed to significant changes in gut morphology. Although there was a numerical increase in villus height in the SDPP group at 12 and 40 days of age, the dietary inclusion of SDPP did not have a significant influence on gut morphometric indices in the birds slaughtered at these time points.

## 6. Dog and Cat Nutrition: SDAP in Pet Food

In the pet food industry, various substances such as proteins, phospholipids, and other surfactants, primarily derived from natural sources, are used as emulsifiers. These ingredients not only act as gelling agents but also undergo evaluations to assess their emulsifying properties. Emulsifiers play a crucial role in achieving desirable texture and stability in pet food products, contributing to their overall quality and palatability. One of the ingredients used as a binder in pet food products is SDAP, characterized by its high protein content, water-holding capacity, foaming and emulsifying properties [85,133], and gel strength when heated above 80 °C [134,135]. Moreover, dogs and cats, as carnivores (facultative and obligate, respectively), require animal products for an optimal diet. SDAP is precisely an additive that is a product of animal origin which may be important for many dog and cat owners looking for products consisting mainly of animal-derived proteins in pet foods.

Quigley et al. [26] described the intake, digestibility, and fecal production of dry dog food diets containing SDAP when fed to adult dogs. Three trials were conducted using 22 beagles, with diets supplemented with different percentages of SDAP. In all three experiments, 2% dietary SDAP raised apparent protein digestibility by 2.2% units and apparent digestibility of dietary dry matter by 2.6%, leading to a 15% decrease in fecal mass. Fecal scores were unaffected by SDAP.

In another dog trial [136] with 36 dogs, extruded food coated with SDPP to levels of 0, 4, 8, or 12% was evaluated. Group-mean apparent total-tract digestibility of CP was 85.2% of intake for the base diet and 85.4, 85.7, and 87.4% for the diets with increasing amounts of SDPP. Apparent DM digestibility was 82.5, 82.8, 83.4, and 87.4%, respectively. Fecal scores were similar for the four diets. SDPP showed high digestibility of CP and DM, making it feasible for use in diets for dogs. However, authors pointed out that it was important to study the effects of inclusion levels lower than 4%, in order not to compromise diet palatability. Similar results were obtained in research on cats, where the addition of SDAP at a level of 30 g/kg to canned food improved the apparent digestibility of DM, crude fiber, ash, calcium, and phosphorus compared to a diet containing wheat gluten [82]. The components in SDAP retained their biological functions even after sterilization and canning, benefiting the digestive system of healthy adult cats.

In a recent study conducted by Lee et al. [137], the effects of diets containing blends of fibers, prebiotics, probiotics, and/or spray-dried plasma on apparent total tract digestibility (ATTD) and stool quality were evaluated in healthy adult intact English pointer dogs. In contrast to the previous study, it was found that the ATTD of DM, organic matter (OM), fat, total dietary fiber (TDF), and energy were lower (*p* < 0.01) in dogs fed a diet enriched with a fiber–prebiotic–probiotic blend (FPPB) or a fiber–prebiotic–probiotic blend with immune-modulating ingredients (i.e., SDAP or iFPPB) compared to those fed a control diet. The ATTD of crude protein (CP) was not affected by the treatment. Additionally, dogs fed FPPB or iFPPB exhibited firmer stools with lower fecal scores (*p* < 0.0001) and higher fecal DM percentage (*p* < 0.0001) compared to those fed the control diet.

Palatability is the measure of intake of a food that indicates acceptance or the measure of preference of one food over another. In the production of commercial pet foods, palatability tests are one of the most important measures to assess the ingredients used in production and their attractiveness to dogs in the final product. Whether the dog eats food with a given additive will also affect the owner, who must be convinced that the food tastes good; otherwise, they will resign from the purchase.

SDAP addition to pet food and its impact on diet palatability were evaluated in a few research studies. Canned, loaf-in-sauce foods, with either SDAP or wheat gluten as the gelling agent, were subjected to two-bowl preference tests involving 20 dogs and 20 cats [134]. The comparisons involved 2% of either agent or 1% SDAP versus 3% wheat gluten. The publication does not show the data, but the authors stated that both the intake and first-choice outcomes were similar within each of the two comparisons, indicating that the dogs did not differentiate between SDAP and wheat gluten. However, cats showed a noticeable preference for the formula containing SDAP. The results indicated that cats enjoyed the taste of plasma and were capable of distinguishing and positively selecting the inclusion of SDAP in the formula, even when it was included at low rates of approximately 10 g/kg.

Another preference test with 20 dogs compared extruded foods with or without SDPP coating [136]. Dogs preferred the 0% SDPP diet over the 4% SDPP diet, suggesting that SDPP in the coating compromised palatability. However, SDPP did not decrease food intake.

Two studies mentioned above applied two-pan tests [134,136], in which dogs had simultaneous access to an excessive amount of two feeds for a limited time. Acceptance of a single product is essential in a home setting. Acceptance equates to willingly eating in sufficient quantity. SDPP in the coating of dry food appeared to reduce the food’s palatability, but it did not decrease food intake [136]. In another dog trial, coating extruded kibbles with SDAP (2% in the final product) also left food intake unchanged [26].

SDAP is widely used as an intestinal health promoter in piglets since it contains active immunoglobulins. It was tested whether an additive to pet food can also affect the health of dogs and cats. Andrade et al. [136] evaluated blood parameters of dogs fed diets containing increasing levels of SDPP. The 12% SDPP diet increased total leukocytes, total blood proteins, and albumin, but the parameters remained within the normal range. Other blood parameters were not affected. In contrast, data from a patent on methods of treatment for modulating the immune system of animals indicate a lack of effect of SDAP intake on white-blood-cell counts after vaccination [138].

In a study in which survival of orally administered porcine immunoglobulins in the gastrointestinal tract of adult dogs and cats fed diets containing spray-dried porcine plasma (SDPP) was measured [139], a level of 1% SDPP reduced fecal excretion of canine IgA in dogs by 41%. The authors suggested that the observed decrease in fecal canine IgA in the dogs fed SDPP was caused by a lower number of intestinal pathogens, which also means that dietary porcine γ-globulins may provide passive immunity in the canine intestine. On the other hand, a lower concentration of IgA in intestinal contents may possibly imply less protection from intestinal pathogens. The study indicated that porcine immunoglobulins partially resist the digestion process in the gastrointestinal tract of adult dogs (8% of immunoglobulins survived) and cats (5%). Similar results were reported in one of the recent studies [137] where fecal IgA concentrations were observed in dogs fed with probiotic- and prebiotic-added (FPPB) or with probiotic-, prebiotic-, and SDAP-added (iFPPB) diets compared to those fed a control diet. This suggests a beneficial immune response to the functional blend, as secretory IgA plays an important role in maintaining gut immune homeostasis. However, the addition of SDAP decreased B lymphocyte populations compared to those fed a control diet but did not alter white blood cells and TNF-α responses. Also, in a study on mice investigating the effects of 0% or 8% dietary inclusion of SDPP to mice’s diet on intestinal microbiome populations, it was found that mice fed with SDPP exhibited a significant rise in microorganisms belonging to the Phylum Firmicutes [140]. This group of microorganisms is known to produce short-chain fatty acids (SCFA), which play a crucial role in regulating the growth of harmful microorganisms and enhancing gut immunity. These findings highlight SDPP’s potential as a prebiotic supplement for promoting gut health and immune tolerance.

Dogs are increasingly being diagnosed with inflammatory bowel disease (IBD), which is the most common cause of intestinal disease and may be associated with parasitosis, food allergies, idiopathic inflammation, or a previously existing disease. The first to discuss IBD in dogs and the use of spray-dried animal plasma to enhance the health of such dogs were Vasconcellos et al. in their recent paper [141]. They noted that while there is no specific study on the effects of SDAP supplementation in dogs with IBD, its various characteristics suggest it could potentially improve the clinical symptoms of the condition, as supplementation with bovine serum immunoglobulin isolate reduced irritable bowel syndrome in humans [104,142]. Further research involving dogs would be needed in this aspect.

SDAP offers satisfying technological properties for pet food production and has been used for many years in dog and cat food products, particularly in chunks and pouch-type products. The inclusion of SDAP not only has a significant positive effect on the texture of pet food chunks but also helps maintain a high level of cohesion among the different ingredients in the recipe. This is due to the high water retention and fat emulsifying capacities of SDAP. Based on the findings of Polo et al. [134], it can be concluded that the presence of more dissociated protein structures and increased separation between highly hydrophilic and lipophilic regions in the polypeptide chain contribute to improved emulsifying properties. SDAP can effectively replace many of the binding agents typically used in wet pet food, offering comparable costs while providing superior technological properties to the final chunk product.

The gelling strength capacity (GSC) refers to the ability of a substance, when dissolved in water, to form a thermoplastic gel under specific conditions such as high temperatures or changes in pH and salt concentration. It is a measure of the product’s capability to undergo gelation and maintain its structural integrity under denaturing conditions.

As reported by Polo et al. [134], the gelling capacity of SDAP is related to the heating temperature (peak values at 121 °C) and the percentage of inclusion of plasma. When SDAP is heated, an irreversible and stable gel is obtained through protein denaturation. Gel formation involves irreversible protein denaturation and interactions between proteins and water, including hydrophobic and electrostatic interactions, hydrogen, covalent, and disulfide bridges. Higher protein concentrations lead to better gelling outcomes due to increased intermolecular contact [134]. SDAP’s ability to form a stable, elastic gel with various advantages makes it useful for food applications, including enhancing texture, water-holding capacity, flavor, nutrient preservation, and minimizing fat losses. In a study evaluating physicochemical and technological properties of different binders used in canned pet food recipes [25], SDAP had the highest GSC among tested binders (wheat gluten, soy protein concentrate, and dehydrated pork rind).

Water-holding capacity (WHC) is a measure of the ability of a powder, such as SDAP, to absorb and retain water after gel formation. To assess this capacity, a gel made from SDAP was prepared by dissolving it in water at concentrations of either 10% or 15%. The gel was then subjected to various temperatures ranging from 70 to 121 °C. Afterwards, the gel was centrifuged at 15,000 rpm for 30 min to separate the released water. The amount of water released from the gels was subsequently analyzed. At the different temperatures tested, the minimum value for WHC was obtained at 80 °C [134], when plasma proteins are denatured. Stronger gels were obtained at 121 °C but with reduced WHC, probably due to a partial disruption of the protein network caused by local aggregation phenomena. According to the authors’ findings in their texture analysis, it was observed that higher concentrations of SDAP resulted in lower water release from the gel at any given temperature. This may be attributed to the increased likelihood of intermolecular contact at higher SDAP concentrations. WHC can be affected by pH [143,144]. WHC and consistency of gels decreases when pH decreases, as reported by Parés et al. [143], where the minimum WHC of their heat-induced gels, from liquid and spray-dried plasma, was at pH 5.5. In a study conducted by Polo et al. [135] to assess the impact of water or poultry fat inclusion in a chunk recipe containing either SDAP or wheat gluten (WG), it was found that the SDAP recipe exhibited less water loss compared to the WG recipe (significant at *p* < 0.05). Additionally, the addition of poultry fat led to an increase in water loss, but acceptable water loss levels were maintained with fat inclusion up to 350 g/kg in the SDAP recipe. SDAP released less water from a centrifuged gel produced at 90 °C than the other binders used in the canned pet food recipes [25].

Fat emulsifying capacity (FEC) refers to the ability of a product to maintain a stable and homogeneous mixture of water and fat (oil). It measures the maximum amount of oil that can be added to an aqueous solution before the emulsion breaks down.

In the case of SDAP, it has been reported to possess a reliable fat emulsion capacity compared to other binders. Studies by Polo et al. [134,135] have demonstrated that SDAP exhibits the highest FEC among various ingredients tested for use as binding or gelling agents in canned pet food. This characteristic is significant for pet food manufacturers as it helps prevent fat exudation in the final products and aids in minimizing variations in fat content among different raw materials used in the recipes.

Due to its high functional qualities (texture, water retention, and emulsifying capacity), SDAP can replace other binding agents at a comparable cost without compromising functionality and may even improve water retention.

## 7. The Additive of SDAP to Aquatic Animals’ Diet

Aquatic animals have distinct and substantial needs for EAAs to support their overall well-being, survival, growth, development, and reproductive processes. As protein is typically the most costly component in aquafeeds, considerable efforts have been focused on ensuring that the dietary protein used for feeding fish, crustaceans, and other aquatic animals is both of high quality and cost-effective. With the rapid expansion of aquaculture on a global scale and limited availability of fishmeal (traditionally the primary source of AAs for aquaculture), there is a growing interest in finding alternative protein sources to meet the nutritional requirements of these animals [145].

Based on the results of several studies, SDAP has shown promising potential as a valuable ingredient in the nutrition of aquatic animals. In largemouth bass (*Micropterus salmoides*), replacing fish meal with SDCP at a rate of 57.14% resulted in increased protein retention, lipid retention, and improved antioxidant capacity, which may contribute to overall health and growth performance [146]. The inclusion of SDCP significantly elevated crude protein content of muscle, liver, and whole fish body. In Pacific white shrimp (*Litopenaeus vannamei*), diets containing 4.5% to 6% SDAP significantly improved growth, feed conversion ratio, immune responses, and resistance to *Vibrio parahaemolyticus* infection [24]. The authors concluded that SDAP could potentially be applied in shrimp farming as an alternative to antibiotics. Nile tilapia (*Oreochromis niloticus*) fed with 49.70 to 51.83 g/kg SDAP exhibited enhanced growth performance, intestinal health, hematological profile, and resistance to cold-induced stress [147], which confirms that improvement of cold resistance in tilapia can be achieved through nutritional modulation. For *Sparus aurata*, SDPP inclusion at 3 g/kg promoted growth and increased the density of intestinal goblet cells [148]. A short administration of SDPP (20 days) resulted in changes in microbiota diversity and richness associated with an increase in the sequences of the genus *Lactobacillus* and to a decrease in the genus *Vibrio*. However, these changes were reversed after 95 days. Interestingly, the density of intestinal goblet cells did not appear to correlate with the changes in microbiota diversity and richness. Instead, it seemed to be influenced more by the immunostimulatory effect of SDPP. In another study on sea bream (*Sparus aurata*), SDPP supplementation at 5% demonstrated positive effects on growth, systemic immunity, and the antibacterial capacity of mucus, suggesting its potential as a functional ingredient in low fish meal aquafeeds [149].

## 8. SDAP and Neuroprotective Effects

Dietary supplementation with SDAP is a common practice in livestock animal production due to its various benefits, including reducing proinflammatory cytokine expression in the gut and promoting growth performance [19]. It has been extensively studied in various research, including rodent models. SDAP has demonstrated anti-inflammatory effects in different inflammation models, such as in the intestinal [150,151,152], pulmonary [153], and genitourinary mucosae in a mouse model. These findings suggest that SDAP may play a crucial role in mitigating the effects of “inflammageing”, the chronic low-grade inflammation associated with aging. As animals age, there is a gradual increase in the production of pro-inflammatory molecules and a decline in the regulatory mechanisms that control the inflammatory response. This chronic inflammation is believed to play a significant role in the aging process and is associated with various age-related diseases and conditions, such as arthritis, immune system dysfunction, and cognitive decline.

There are reports suggesting that the addition of SDAP to the diet can prevent cognitive impairment. Researchers conducted a study to investigate whether the systemic anti-inflammatory effects of SDAP could extend to non-mucosal tissues such as the brain [154]. Specifically, they wanted to examine the potential impact of SDAP, known for its anti-inflammatory and antioxidant properties, on cognitive functions and brain integrity markers in a mouse model of accelerated senescence (SAMP8). The hypothesis was that SDAP, as a dietary supplement capable of controlling peripheral mucosal inflammation, might also have beneficial effects on brain regions that are connected to the gut. Age-related cognitive decline is often accompanied by an inflammatory profile [155], and a dietary supplement that can control peripheral mucosal inflammation should also be helpful in brain regions interconnected with the gut [156,157]. The study confirmed that SDAP supplementation not only improved cognitive deterioration but also attenuated age-related changes in brain markers of inflammation and oxidative stress. These findings indicated the neuroprotective effects of SDAP in the senescent SAMP8 mice [154]. Furthermore, the results showed a significant decline in short- and long-term memory in 6-month-old animals, which was completely prevented by SDAP supplementation. Another study concluded that dietary SDAP might delay Alzheimer’s disease onset by reducing its hallmarks in senescent mice [158]. In a recent study by Rosell-Cardona et al. [157], SDAP supplementation reduced neuroinflammation and improved cognitive performance. Senescent mice exhibited impaired memory and altered cytokine levels, which SDAP mitigated.

While the use of animal blood in animal feed has been investigated for a long time, and safety concerns have been addressed [159], the consumption of animal blood in human nutrition remains controversial. However, the research results cited above may be applicable to carnivorous pet animals, for which the consumption of blood products is normal and even desirable. These animals, living longer and longer, may face problems with a decline in physical or mental health, which can be a challenge for the owners [160]. With increasing age, dogs develop a form of neurodegenerative disease, which has many similarities to age-related cognitive impairment and Alzheimer’s disease in humans [161]. Therefore, it seems quite likely that the addition of SDAP to the diet of aging pets could provide similar benefits to those obtained in studies on mice. However, this requires careful examination and confirmation of the results.

## 9. Conclusions

In conclusion, the reviewed scientific literature highlights that spray-dried plasma is a unique functional ingredient with significant benefits for intestinal health and functionality. It achieves this by modulating both the local and systemic immune systems. The literature examined the effects of SDAP on various livestock species and also discussed the health advantages it provides to pets. Overall, the evidence suggests that SDAP enhances intestinal health and promotes an efficient immune system response at both local and systemic levels. This helps reduce the diversion of nutrients for immune system activation, allowing them to be utilized for productive functions instead. As a result, the inclusion of SDAP in animal diets leads to improved overall performance and well-being. SDAP acts as a valuable ingredient that enhances intestinal health, which, in turn, positively affects digestive metabolism and animal growth performance. Additionally, there is evidence to suggest that SDAP supplementation may have the potential to prevent cognitive impairment, which is particularly relevant for senior dogs and cats.

## Data Availability

No new data were created or analyzed in this study. Data sharing is not applicable to this article.
